# The Hospital World

**Published:** 1910-04-30

**Authors:** 


### THE HOSPITAL WORLD.

The King of the Belgians has accepted the patronage of the Finsbury Dispensary, now in its 129th year.

The Hon. W. F. D. Smith has become a Vice-President of the Margate Royal Sea Bathing Hospital for surgical tuberculosis.

The Earl of Leicester has accepted the presidency of the Norfolk and Norwich Hospital, and succeeds the late Earl in that office.

Lord Ancaster has resigned the office of President of the Evelina Hospital for Sick Children. The Court of Governors, therefore, has chosen Lord Duncannon, M.P., to succeed him.

Sir William Watson, D.L., has been elected a vice-president of the Rotunda Hospital, Dublin. A resolution has been proposed and seconded that Mr. F. D. Darley be elected a governor at the next Charter Board meeting.

Major-General Edmeades, J.P., Mr. H. B. Hohler, D.L., J.P., and Mr. W. Tingey, J.P., have been nominated Vice-Presidents of the Gravesend Hospital. Mr. H. Golding-Bird and the Mayor, Mr. Alderman H. E. Davis, have become Life Governors.

Sir Allen Young, C.B., C.V.O., the intrepid explorer and navigator, has just been elected Vice-President of the Seamen's Hospital Society. Sir Allen Young served as a member of the Committee of Management of the "Dreadnought" from 1862 to 1866, not long after his return in the "Fox" from the successful search for the records of Franklin's missing ship.

The Herefordshire General Hospital has re-elected its Board of Management as follows: Lieut.-Gen. Sir Edward Hopton, K.C.B., the Hon. and Very Rev. the Dean of Hereford, the President, the Vice-Presidents, the Earl of Chesterfield, Lord Biddulph of Ledbury, Sir H. Archer Croft, Bart., Sir J. R. G. Cotterell, Bart., Sir Geoffrey Cornewall, Bart., Sir Joseph Verdin, Bart., Mr. J. S. Arkwright, M.P., Dr. W. F. Abbott, Mr. E. Bellerby, Mr. C. P. Bird, Colonel R. Bourne, Mr. H. P. Bulmer, Mr. J. U. Caldicott, Mr. W. T. Carless, Major A. Chambers, Mr. G. Child, Captain Percy A. Clive, M.P., Mr. George Cresswell, Mr. W. F. Dury, Mr. A. W. Foster, Colonel Hewat, Mr. R. T. Hinckes, the Rev. Preb. M. Hopton, Mr. W. E. King-King, Colonel O. R. Middleton, Captain T. L. Morgan, the Rev. J. C. Murray-Aynsley, Mr. H. Ronalds, Mr. A. Simpson, Mr. R. H. Symonds-Tayler, Mr. Philip F. Walker, Mr. Witts. The working men's representatives on the Board of Management are Mr. A. Adkins and Mr. W. Ashburner.

Mr. W. E. Green, whose generosity to the Kingston Hospital has been continual, has been rewarded by the Corporation with admission as Honorary Freeman of the Borough. Before 1906 he paid the expenses of re-decoration throughout the building, and made that year an *annus mirabilis* to the hospital by doubling its accommodation. This extension led, as always, to the need for an endowment fund, which Mr. Green started with £250, and Mrs. Green has seconded him by challenging the neighbourhood to raise five thousand shillings, which she herself will equalise by a collection from her friends in London. The response this challenge has received already shows that the spirit of personal service in the borough has permeated all those who are working for the hospital in the generous rivalry it has awakened.

At the last election of directors of the Royal Alexandra Infirmary, Paisley, Scotland, the subscribers' votes declared for Mr. John A. Brown, Mr. G. H. Coats, Mr. Thomas Greenlees, Mr. George Hamilton, Mr. George Hart, and Mr. John Millar, who accordingly have become directors.

The Committee of Management of the Colwyn Bay and District Cottage Hospital has been re-elected as follows:—The Rev. John Griffiths (Vicar of Colwyn), the Rev. T. M. Jones, Mr. J. W. Raynes, J.P., Mr. W. L. Whitehouse, Mr. H. Charlton Jones, Mr. F. Stancliffe, Mr. David Lewis, Mr. Joseph Smith (Parciau), and Mr. Francis Nunn.

The Hertford and Ware Joint Hospital Board has lost two useful members apparently as the result of local political changes. At a recent meeting the Chairman of the Board, Mr. W. F. Andrews, said he would like to point out that Mr. Catling, who was appointed Vice-Chairman at the last meeting, had written to say he was sorry he had to retire from the Board, as he had lost his seat on the Ware Urban Council. "I had another letter from Mr. Horsey," Mr. Andrews went on, "saying that after twelve years' service he had lost his seat on the Ware Board of Guardians, and he had to retire. He thanked the members, and especially the late Chairman, for the great courtesy he had received from them. I suppose this is the result of the fortunes of war, but unfortunately we have lost two useful members. As far as the Vice-Chairman is concerned, we shall have to consider whether we will appoint his successor now or put the matter on the agenda for the next meeting." It was decided to defer the appointment. Mr. Horsey had been a most useful supervisor of the expenditure and accounts, and Mr. Catling, who lived nearer than anyone else to the hospital, had gone backwards and forwards on many occasions when he had not been called upon to do so. Mr. Ashwell proposed that the Clerk should send letters of regret at the retirement of these members from the Board, and this was agreed to.

A recent meeting of the governors of Lincoln County Hospital concerned itself with the proposal to appoint a new officer—a secretary-superintendent. The proposal has been the outcome of a committee, the aim of which was to inquire into the existing state of affairs and to point out any improvements that were likely to increase the efficiency of the institution. The proposal was described by Canon H. W. Hutton (the Chairman) as "one of the greatest alterations or reforms that has come before the Board for a great number of years." Mr. A. H. Leslie-Melville pointed out that it was a universal custom for hospitals to have a secretary-superintendent. At Lincoln the secretary's work has been done hitherto by five different persons, chief perhaps of whom has been Mr. Danby, who, with Mr. Watkins, the surveyor, was congratulated warmly by the Board for his services, and those of his clerks. The matron, too, has been much occupied with bookkeeping. Some discussion took place on the amount of salary to be given to the new officer, and it is probable that £250 will be granted, though Mr. W. S. Richardson suggested that that sum was too small. The appointment, we understand, has not yet been filled. Mr. W. B. Danby has been secretary to the Board of Governors for thirty-six years, and in the last few years a new lavatory has been built and the establishment increased in a variety of ways, which has added to his work in many directions.

